# Navigating the Endodontic Challenge of a 40 mm Canine and Its Technical Adaptations

**DOI:** 10.1155/2024/9962576

**Published:** 2024-06-24

**Authors:** Siavash Moushekhian, Pooya Saeedi, Zoha Sahebnasagh

**Affiliations:** ^1^ Department of Endodontics School of Dentistry Mashhad University of Medical Sciences, Mashhad, Iran; ^2^ Independent Researcher, Mashhad, Iran

## Abstract

*Aim*. The aim of this case report is to present the successful endodontic management of an abnormally long right maxillary canine diagnosed with symptomatic irreversible pulpitis, highlighting the technical adaptations employed during treatment. *Summary*. Technical adaptations included modifying the handle of the endodontic hand file and utilizing a side-vented microcannula for irrigation using positive and negative pressure technique. Despite the challenges posed by the abnormal tooth length, the procedure achieved a working length of 40.7 mm, and periapical radiography confirmed the master gutta-percha reaching full working length. Notably, the patient remained asymptomatic during the six-month follow-up, highlighting the efficacy of the treatment. This article also reviews the current literature, examining comparable cases and techniques documented in scholarly sources.

## 1. Introduction

Understanding the anatomy of the root canal system and its morphological variations is crucial for the success of every step of endodontic treatment. Comprehensive knowledge of the intricate details of the root canal's anatomy enables clinicians to employ the most suitable treatment techniques and protocols, ultimately enhancing the overall success rate of the procedure [1]. The complex nature of canal systems poses new challenges, underscoring the importance of thorough disinfection [2]. Effective disinfection is particularly vital because any debris that remains adhered to canal walls after mechanical instrumentation can compromise the cleaning process, leading to potential treatment failures [3].

Achieving proper instrumentation of the root canals and ensuring complete debridement are key objectives in endodontic procedures [4]. However, a notable challenge arises in the case of long canals, where reaching the total length of the tooth can be difficult [5]. Uncommonly encountered, extremely long teeth, also referred to as radiculomegaly, have rarely been documented in the literature, posing a distinctive challenge in achieving thorough mechanical cleaning of the root canal. The canine, particularly the maxillary canine, is the most commonly affected tooth in this context, with a typical average length of 27.3 mm [6, 7].

Several studies have documented the extraction of maxillary canines with exceptionally long roots, reaching up to 52 mm in length [8–12]. Moreover, various studies have investigated the root canal treatment procedure and necessary modifications for such lengthy roots [13–21]. Given the inherent difficulty in performing endodontic procedures and cleaning the entire root length, compounded by the unavailability of commercially produced endodontic instruments longer than 31 mm, our case report is aimed at detailing the biomechanical preparation and obturation of an unusually long maxillary canine measuring 40.79 mm. In this report, we seek to explore and discuss the treatment modifications essential for the successful management of these exceedingly rare cases.

## 2. Report

This case report was written according to the Preferred Reporting Items for Case Reports in Endodontics (PRICE) 2020 guidelines. In this case study, a 32-year-old male patient with a noncontributing medical history sought treatment at a private endodontic dental office. The chief complaint was sharp and lingering pain upon thermal stimuli, often persisting for 30 seconds or longer after the stimuli stopped. Additionally, the patient reported occasional spontaneous pain in the right canine and no discomfort during biting.

**Figure 1:**
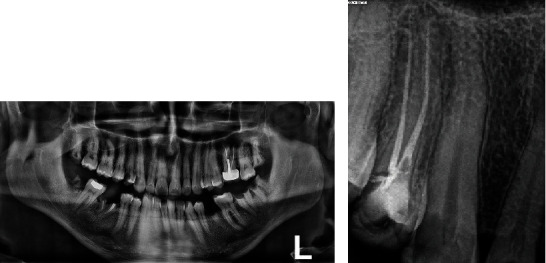
Pretreatment. (a) Panoramic and (b) periapical radiographs.

Clinical examination revealed a wide carious distal wall. Intense pain was elicited during thermal testing and electronic pulp testing, but there was no tenderness upon percussion or palpation. A periapical and panoramic radiograph indicated an intact lamina dura, a uniform periodontal ligament space, and an abnormal length in the suspected tooth (#13) (Figure 1). No abnormalities were detected during the extraoral examination.

Based on the clinical and radiographic findings and adhering to the guidelines of the American Association of Endodontics (AAE) 2013, the patient was diagnosed with symptomatic irreversible pulpitis with normal apical tissue. The chosen treatment plan involved nonsurgical primary endodontic treatment.

**Figure 2:**
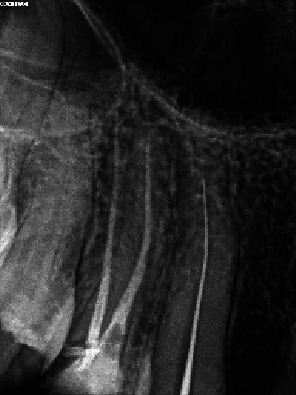
Periapical radiograph indicating a working length of 40 mm and the need for an additional 9 mm to achieve full working length.

Endodontic treatment was performed by an experienced endodontic specialist (S.M.). The procedure commenced with the administration of infiltration anesthesia, utilizing 2% lidocaine with 1 : 100,000 epinephrine (Xylopen 2%, Exir Pharmaceutical Co., Tehran, Iran), and isolation with a rubber dam (Henry Schein Inc., Melville, NY, USA). Afterwards, the carious tissue was removed from the distal wall, and the access cavity was prepared under magnification using a dental operating microscope (DOM) (3200 R2/PRO, Zumax Medical Co., Jiangsu Province, China). Canal coronal flaring was achieved using Gates-Glidden sizes 1-3 (MANI Inc., Tochigi, Japan). The initial estimated working length was determined to be 38 mm based on an OPG. A 31 mm #15 K-file (MANI Inc., Tochigi, Japan) without a rubber stop was then inserted to its full length. A periapical radiograph was taken to evaluate the necessary modifications, confirming a working length of 40 mm and indicating a need for an additional 9 mm (Figure 2).

**Figure 3:**
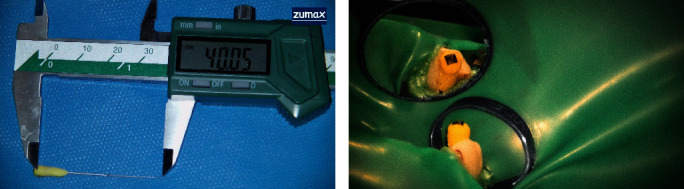
Modified handle of the endodontic hand file, prepared with diamond burs. (a) Endodontic hand file measured with a digital caliper. (b) File inserted into the tooth at its full working length.

**Figure 4:**
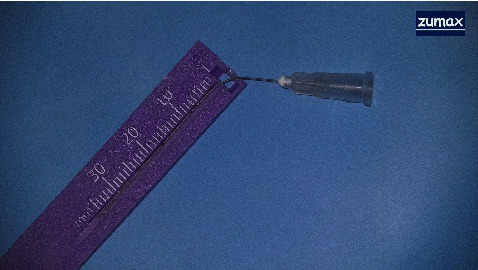
The 50 mm side-vented 27-gauge microcannula used for irrigation.

To achieve the desired length, the handle of the endodontic hand file was carefully modified using diamond burs (Figure 3). The canal was instrumented with a passive step-back technique, employing modified hand files (initial file #15, master file #30). Root canal preparation was conducted with irrigation using 2.5% sodium hypochlorite (CERKAMED, Stalowa Wola, Poland). A 50 mm side-vented 27-gauge microcannula (Time Machine Co., Gyeonggi, Korea) (Figure 4) was employed, utilizing a positive and negative pressure technique after each file change. After completing the preparation and instrumentation phase, active irrigation was performed using a sonic device (EDDY, VDW, Munich, Germany). The canal was filled to the orifice with 5% sodium hypochlorite and activated for 1 minute. Following a rinse with distilled water, 17% EDTA (CERKAMED, Stalowa Wola, Poland) was similarly activated. A final rinse with distilled water was conducted before proceeding with obturation.

**Figure 5:**
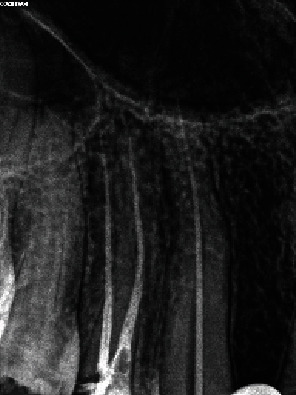
Periapical radiograph confirming master gutta-percha reaching the full working length.

**Figure 6:**
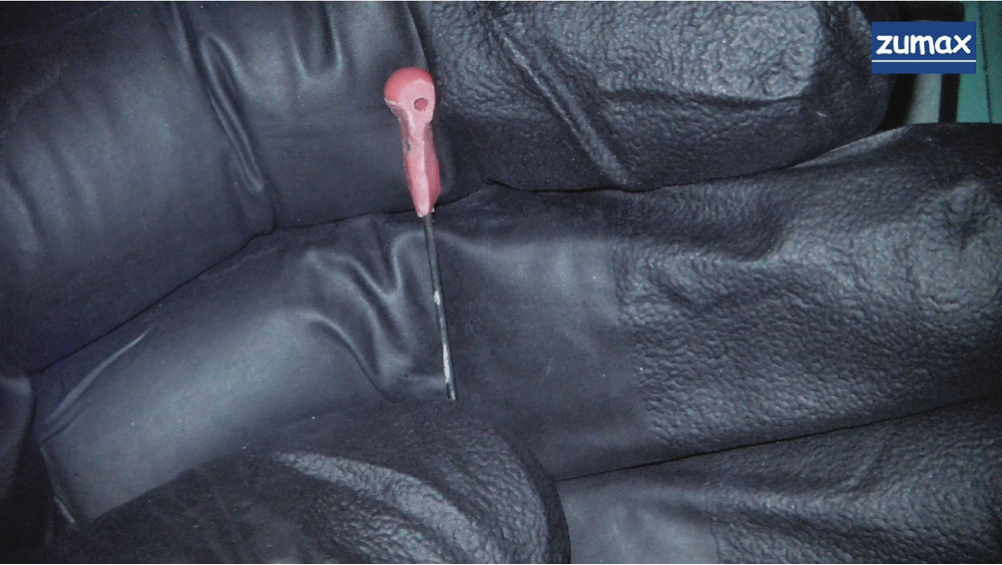
Modified handle of the spreaders.

**Figure 7:**
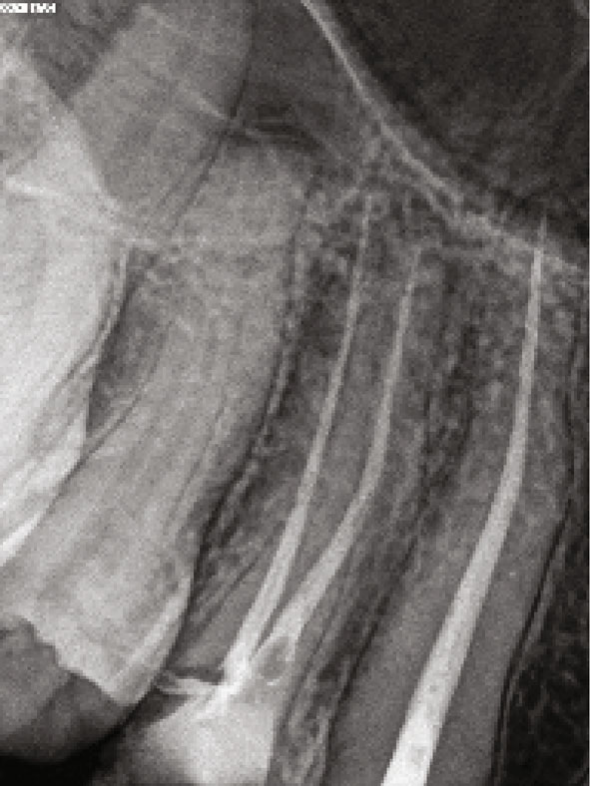
Periapical radiograph following the completion of root canal treatment.

Following thorough drying of the canal with endocanal suction (Arad Biomed, Mashhad, Iran) using a 50 mm side-vented 27-gauge microcannula, which effectively dried the apical area, along with paper cones (#30), root canal filling was initiated. A master gutta-percha (#30) (DiaDent Group International, Korea) was placed at its full length. Due to the shorter length of the gutta-percha compared to the canal length, the reference point for the gutta-percha was marked within the canal, just below the cementoenamel junction (CEJ), visible only under a DOM. Confirmation was obtained through a periapical radiograph (Figure 5). The cold lateral filling technique with an AH Plus sealer (Dentsply Sirona, Charlotte, NC, USA) and modified 31 mm spreaders (MANI Inc., Tochigi, Japan) (Figure 6) was employed, and excess material was carefully removed using a heated gutta-percha condenser (Fast-Pack Pro, Eighteeth, Changzhou City, Jiangsu Province, China). Before placing a temporary restoration using Cavit (Coltosol, Asia Chemi Teb Co., Tehran, Iran), the pulp chamber was thoroughly cleaned. Another periapical radiograph was taken after the completion of root canal treatment (Figure 7).

**Figure 8:**
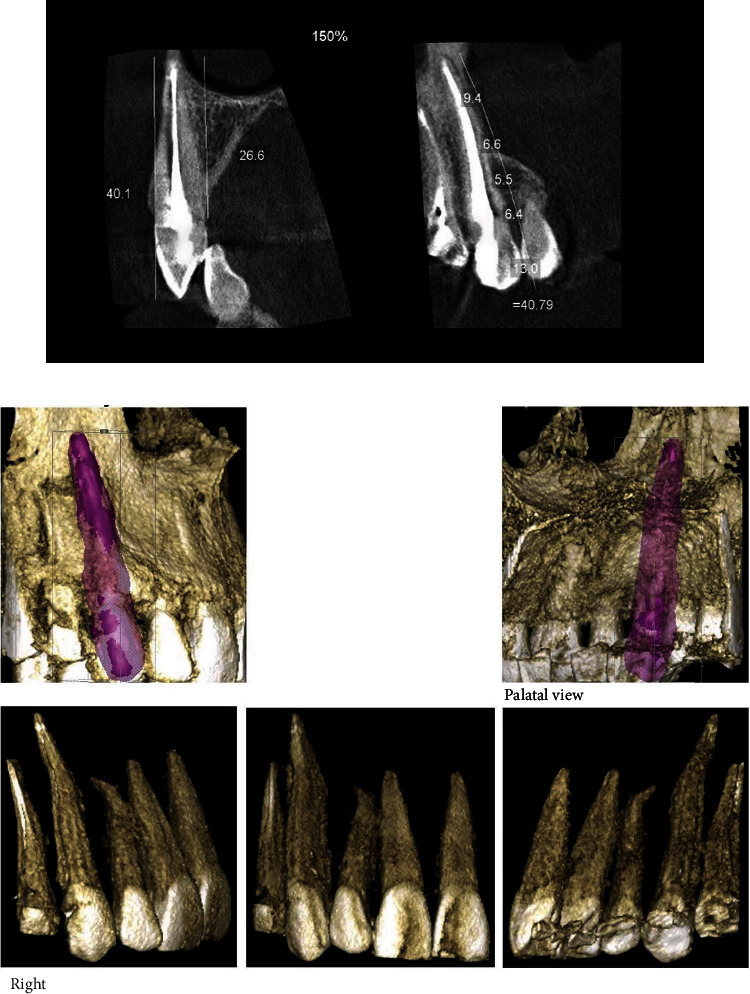
Posttreatment CBCT radiographs in six-month follow-up. (a) Coronal (left) and sagittal (right) slices. (b) 3D model of the treated tooth.

**Figure 9:**
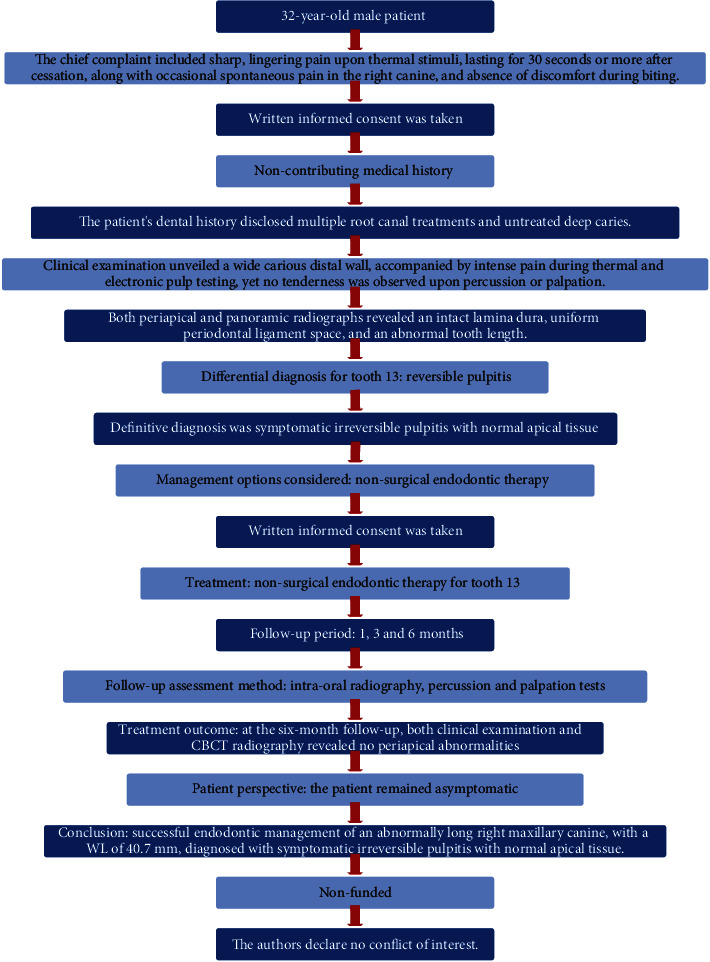
PRICE 2020 flowchart.

At the six-month follow-up, both clinical examination and CBCT radiography revealed no periapical abnormalities (Figure 8). The patient remained asymptomatic, indicating a successful posttreatment outcome.  Figure 9 displays an overview of the case report using PRICE 2020 flowchart.

## 3. Discussion

Ensuring precise locating, thorough cleaning, proper shaping, and effective obturation of the root canal system is crucial for the long-term success of root canal therapy. Deviations from any of these fundamental principles may risk treatment outcomes, leading to posttreatment diseases, pain, or complications associated with the treated tooth [1, 22].

Accurate determination of the working length is essential for successful root canal therapy and plays a key role in minimizing the risk of inadequate canal cleaning or periapical tissue damage resulting from overinstrumentation [23]. The atypical length of human teeth appears to be unrelated to the individual's stature, as evidenced by our patient's height of 180 cm. Wilkie and Chambers asserted that tooth length does not necessarily correlate with the patient's height, underscoring the importance of obtaining high-quality radiographs prior to tooth extraction or endodontic treatment [11].

Recognized as the longest teeth in the human dental arch, upper cuspids feature a flattened cervical region that requires flaring for adequate instrumentation. Additionally, they typically present a single wide canal with greater buccopalatal and lower mesiodistal dimensions, facilitating the endodontic treatment process. However, the unique lengths of cuspids, which rarely exceed 31 mm, pose technical challenges, necessitating innovative approaches by professionals to prevent crown wear.

The challenges associated with the instrumentation of upper cuspids, particularly those exceeding 31 mm in length, are noteworthy. The size limitations of commercially available instruments make the procedure exceptionally difficult. Despite these challenges encountered in clinical practice, our discussion highlights an alternative treatment technique applied in a specific case. This technique not only preserves the crown but also has potential utility in addressing similar cases.

In a review of the literature addressing the challenges associated with treating long teeth, four distinct strategies have been identified to overcome the limitations of conventional endodontic instruments. These strategies include (1) endodontic instrument modification [15–17], (2) altering the reference point of the endodontic instrument [13, 14, 20], (3) selectively wearing the crown with a drill to achieve the ideal working length, and (4) employing veterinary files.

These strategies, discussed individually or in combination, are chosen based on the specific challenges posed by each case. In the context of this report, the selected procedure involved endodontic instrument modification. Endodontic instrument adaptation entails thinning approximately 8 mm from the handle of the endodontic hand file using a diamond bur, providing additional length up to 40 mm during root canal preparation to reach the desired working length. Cardoso et al. [16] emphasized the need to reduce rotational movement amplitude and apical force when employing this technique. Hussain and Awooda [17] suggested cable removal by heating it over a flame until it is soft, allowing it to be pulled away by dental tweezers. Hand files are then manipulated with an inverted pen grasp using a push-pull movement for biomechanical preparation.

Barletta et al. [14] described a case involving a 36 mm maxillary canine, where the reference point of the endodontic instrument transitioned from the incisal to the cervical limit. With K-files, the root canal preparation left a 1.5 mm gap between the file tip and the radiographic apex (34.5 mm working length). Changing the reference point to the palatal aspect allowed a 12 mm reduction in the overall working length, making 31 mm files suitable for effective root canal cleansing and shaping [20]. In cases requiring further adjustment, endodontists may resort to selectively wearing the crown with a drill to reach the ideal working length, although this approach is recommended only for minor modifications due to its potential for crown damage and weakening.

Shifting the reference point of the endodontic instrument appears to be a safer alternative, as it preserves the physical characteristics of the instrument while offering greater length gain, as noted by Vargo and Hartwell [20]. Furthermore, these clinical techniques can be synergistically employed. Another viable option involves the use of veterinary files, such as the Vetinox® Hedstroem File (Dentsply Maillefer, Ballaigues, Switzerland), with lengths of 40 and 60 mm, serving as an alternative solution for such challenging cases. Maden et al. [18] and Pace et al. [19] presented cases of successful root canal treatments using these files for a mandibular canine and mandibular first premolar, respectively. However, in Iran, obtaining the Vetinox line has proven challenging, leading to its exclusion from consideration for use.

Selective crown wear by drilling to achieve the ideal working length is advised only for minor adjustments, given the potential for crown damage and weakening. In the present case, this strategy was not employed, as the desired working length was successfully achieved using the aforementioned techniques.

Upon reviewing this clinical case alongside other studies on dental anatomy, the necessity for longer endodontic instruments in the market becomes apparent. Cuspids exhibit lengths that can surpass the maximum capacity of currently available endodontic instruments. Consequently, situations of extreme length may arise. In the absence of viable alternative techniques, there is a potential risk of resorting to wearing down the coronary structure or, in extreme cases, losing a dental element.

However, it is important to note the relatively short follow-up period as a limitation of this case report, warranting further long-term observation to assess treatment durability and success over time.

## 4. Conclusion

Performing root canal treatment on patients with exceptionally long teeth presents a challenge. However, this report demonstrated that employing the specified clinical methods can effectively instrument these patients endodontically. Practitioners need to be prepared to handle long teeth in their clinical work, and the industry should explore additional endodontic instrument options.

## Data Availability

The data that support the findings of this study are available upon request from the corresponding author.

## References

[1] Vertucci F. J. (2005). Root canal morphology and its relationship to endodontic procedures. *Endodontic Topics*.

[2] Siqueira J. F., Alves F. R., Almeida B. M., de Oliveira J. C., Rôças I. N. (2010). Ability of chemomechanical preparation with either rotary instruments or self-adjusting file to disinfect oval-shaped root canals. *Journal of Endodontia*.

[3] Dotto L., Sarkis Onofre R., Bacchi A., Rocha Pereira G. K. (2020). Effect of Root canal irrigants on the mechanical properties of endodontically treated teeth: a scoping review. *Journal of Endodontics*.

[4] De-Deus G., Barino B., Zamolyi R. Q. (2010). Suboptimal debridement quality produced by the single-file F2 ProTaper technique in oval-shaped canals. *Journal of Endodontia*.

[5] De-Deus G., Reis C., Beznos D., de Abranches A. M., Coutinho-Filho T., Paciornik S. (2008). Limited ability of three commonly used thermoplasticized gutta-percha techniques in filling oval-shaped canals. *Journal of Endodontia*.

[6] Kuzekanani M., Jafari A. M. (2020). Root canal anatomy and morphology of permanent maxillary canine teeth in an Iranian population. *Italian Journal of Anatomy and Embryology=Archivio Italiano di Anatomia ed Embriologia*.

[7] Bjorndal A. M., Henderson W. G., Skidmore A. E., Kellner F. H. (1974). Anatomic measurements of human teeth extracted from males between the ages of 17 and 21 years. *Oral Surgery, Oral Medicine, Oral Pathology*.

[8] Hayward J. R. (1980). Cuspid gigantism. *Oral Surgery, Oral Medicine, and Oral Pathology*.

[9] Marashi A. H., Gorlin R. J. (1990). Radiculomegaly of canines and congenital cataracts--a syndrome?. *Oral Surgery, Oral Medicine, and Oral Pathology*.

[10] Booth J. M. (1987). The longest tooth?. *Australian Endodontic Newsletter*.

[11] Wilkie G. J., Chambers I. G. (1990). A very large maxillary cuspid. *Oral Surgery, Oral Medicine, and Oral Pathology*.

[12] Weine F. S. (1986). A very long cuspid!. *Journal of Endodontia*.

[13] Al-Dahman Y., Al-Hawwas A., Al-Jebaly A. (2017). Root canal treatment of a 32-mm length maxillary canine - a case report. *International Journal of Contemporary Medical Research*.

[14] Barletta F. B., Grecca F. S., Wagner M. H., Ferreira R., López F. U. (2010). Endodontic treatment of a 36-mm long upper cuspid: clinical case report. *Revista Odonto Ciência*.

[15] Bellizzi R. (1982). Endodontic therapy associated with a case of cuspid gigantism. *Oral Surgery, Oral Medicine, and Oral Pathology*.

[16] Cardoso R. M., Vieira T. M., Limoeiro A. G., Bastos H., Tomazinho L. F., Albuquerque D. S. (2019). An alternative technique to Endodontic treatment for long teeth: a case report. *Journal of Surgical and Clinical Dentistry*.

[17] Hussain S. E., Awooda E. M. (2020). Root canal treatment for a lengthy maxillary canine of 37 mm. *Journal of Case Reports in Dental Medicine*.

[18] Maden M., Savgat A., Görgül G. (2010). Radiculomegaly of permanent canines: report of endodontic treatment in OFCD syndrome. *International Endodontic Journal*.

[19] Pace R., Giuliani V., Pagavino G. (2011). Endodontic management in oculo-facio-cardio-dental syndrome: a case report. *Journal of Endodontia*.

[20] Vargo J. W., Hartwell G. R. (1992). Modified endodontics for lengthy canals. *Journal of Endodontia*.

[21] Venokur P. C., Fink H. D. (1976). Maxillary canine of unusual length. *Oral surgery, oral medicine, and oral pathology*.

[22] Sjogren U., Hagglund B., Sundqvist G., Wing K. (1990). Factors affecting the long-term results of endodontic treatment. *Journal of Endodontia*.

[23] Naulakha D., Agrawal M., Naulakha N. (2015). Determination of tooth length variation of maxillary canine - an analytical study. *Journal of Nobel Medical College*.

